# The Impact of Blood Rheology on Drug Transport in Stented Arteries: Steady Simulations

**DOI:** 10.1371/journal.pone.0128178

**Published:** 2015-06-12

**Authors:** Pujith R. S. Vijayaratnam, Caroline C. O’Brien, John A. Reizes, Tracie J. Barber, Elazer R. Edelman

**Affiliations:** 1 School of Mechanical and Manufacturing Engineering, the University of New South Wales, Sydney, New South Wales, Australia; 2 Harvard-MIT Biomedical Engineering Center, Massachusetts Institute of Technology, Cambridge, Massachusetts, United States of America; Texas A&M University, UNITED STATES

## Abstract

**Background and Methods:**

It is important to ensure that blood flow is modelled accurately in numerical studies of arteries featuring drug-eluting stents due to the significant proportion of drug transport from the stent into the arterial wall which is flow-mediated. Modelling blood is complicated, however, by variations in blood rheological behaviour between individuals, blood’s complex near-wall behaviour, and the large number of rheological models which have been proposed. In this study, a series of steady-state computational fluid dynamics analyses were performed in which the traditional Newtonian model was compared against a range of non-Newtonian models. The impact of these rheological models was elucidated through comparisons of haemodynamic flow details and drug transport behaviour at various blood flow rates.

**Results:**

Recirculation lengths were found to reduce by as much as 24% with the inclusion of a non-Newtonian rheological model. Another model possessing the viscosity and density of blood plasma was also implemented to account for near-wall red blood cell losses and yielded recirculation length increases of up to 59%. However, the deviation from the average drug concentration in the tissue obtained with the Newtonian model was observed to be less than 5% in all cases except one. Despite the small sensitivity to the effects of viscosity variations, the spatial distribution of drug matter in the tissue was found to be significantly affected by rheological model selection.

**Conclusions/Significance:**

These results may be used to guide blood rheological model selection in future numerical studies. The clinical significance of these results is that they convey that the magnitude of drug uptake in stent-based drug delivery is relatively insensitive to individual variations in blood rheology. Furthermore, the finding that flow separation regions formed downstream of the stent struts diminish drug uptake may be of interest to device designers.

## Introduction

Although blood is a non-Newtonian fluid, flow in stented arteries has often been modelled numerically with a Newtonian blood model [1–3]. In order to capture the shear-thinning properties of blood, some computational studies of stented arteries [4–6] have attempted to more accurately model blood flow through the implementation of non-Newtonian blood rheological models [7–10]. Despite the fact that each model has been created by parameter-fitting to experimental measurements of blood viscosity, they vary widely in the prediction of the blood viscosity at the same strain rates. As blood viscosity not only differs significantly between males and females [11], but also between persons of the same sex [11], it is unlikely that any rheological model can be developed which would capture the highly variable rheological properties of blood.

One of the primary causes of these variations is the hæmatocrit [9], usually defined as the ratio of the volume taken up by red blood cells to the total volume of blood. Phillips *et al*. [12] showed that the hæmatocrit decreases significantly in the aftermath of the angioplasty procedures used in stent implantation, likely from blood loss and fluid resuscitation. They found that whereas the average hæmatocrit prior to the operation had been 40% in men and 38% in women, in the 12 hours following the procedure these values dropped to 34% and 33% respectively. The Carreau non-Newtonian blood rheological model has been utilised in some past numerical analyses of stented arteries [4, 5] but unlike some other rheological models, such as the Walburn-Schneck and Casson models, it cannot simulate the effects of differences in hæmatocrit. Hence, past analyses of steady-state drug deposition have not taken into account the effect of the reduced hæmatocrit on the resulting blood flow. It is important to characterise these post-angioplasty reductions in hæmatocrit to better predict the early ‘burst’ release of drug from strut coatings, which can take place within the first few days of stent implantation [13].

Following our previous work [5], the same computational model is used to explore the factors governing the fluid dynamic environment within the vasculature and their effects on drug distribution patterns. We also previously used a custom-designed bench-top experiment consisting of a single drug-eluting stent (DES) strut and tissue bed with a Newtonian working fluid to validate the computational findings and showed experimentally that pulsatile flow only has a small effect on drug transport when the strut is well-apposed. This was in qualitative agreement with numerically generated data, thereby justifying the use of steady-state simulations, which are simulations in which all flow parameters remain constant with respect to time. A series of these steady-state computational fluid dynamics (CFD) analyses are performed in the present study with the primary aim of determining the impact of different blood rheological models on the haemodynamic flow details and drug transport behaviour. We anticipate only subtle changes in drug delivery on account of rheology based on earlier studies [4], which may be obscured or masked in in-vitro or animal models. The use of numerical methods represents the ideal platform in which to study the impact of rheology owing to the multiscale resolution of the phenomena in the computational domain and the tight control on boundary conditions. The numerical results obtained with the traditional Newtonian viscosity model are compared with data generated with the Power Law, Walburn-Schneck, Casson, Carreau and Generalised Power Law non-Newtonian viscosity models, chosen because they span the clinical spectrum of apparent viscosity variations. These results are further compared against those obtained with an additional model with the fluid properties of plasma to ensure that the full range of near-wall blood behaviours are modelled. A secondary objective is to determine the effect of changing the hæmatocrit on the flow details and drug transport, so as to determine whether the post-angioplasty reduction in hæmatocrit results in significant changes in the flow and drug transport behaviour of the stent.

## Materials and Methods

### Geometry

**Fig 1:**
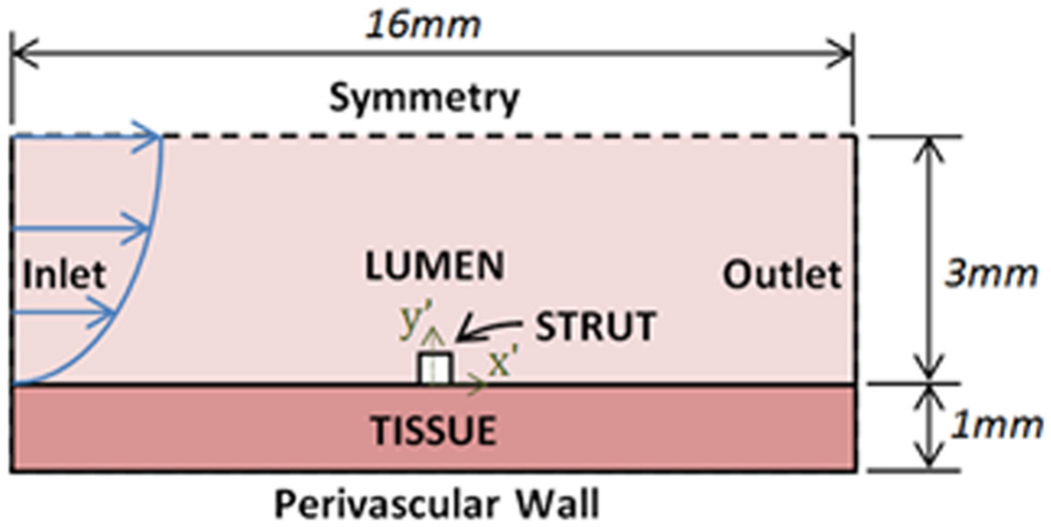
Schematic Diagram of Geometry: Dimensions and Boundary Conditions.

This two-dimensional numerical study of blood flow in a stented artery encompassed the modelling of the flow field and drug concentration distribution in the artery lumen, as well as the drug concentration distribution in the arterial tissue. The lumen was modelled as a 3mm radius fluid domain, whilst the tissue was modelled as a 1mm thick, homogeneous, porous cylindrical tube. A single 0.1mm square cross-section DES strut was also modelled halfway between the inlet and outlet of the computational domains, as may be seen in Fig 1. These dimensions are identical to those used in our prior analysis of stent-based drug therapy in the renal vasculature [5] and represents the application of established numerical techniques [1,4] to implants in non-coronary vasculatures, reflecting current clinical interest [14–17].

We constructed an idealised geometry that still captured the breadth of flow features present in 3D complex geometries. This included a single stent strut obstructing near wall flow and generating strut adjacent recirculation regions where drug can pool [18]. A more realistic model of 3D implants would likely consider multiple struts. When considered in this context, upstream struts act to shield downstream struts from flow, and superposition of drug occurs [1]. Flow-mediated drug uptake was always most significant at the foremost proximal strut—where the flow disruptions were most significant and the contribution from neighboring struts was quite small [1]. Selecting then a geometry that most significantly enhanced the sensitivity of drug uptake to arterial flow changes, we refined the geometry to be a single-stent strut isolated in the boundary layer of flow. A square edge was similarly chosen in place of rounded or chamfered edges to yield the most exaggerated flow field.

### Mathematical Model

In steady incompressible flow the equations of conservation of mass and momentum are written:

$$\nabla \prime \cdot {\text{v}}_{l}\text{'}=0$$ (1)

and

$$\rho \prime \left[{\text{v}}_{l}\prime \cdot \nabla \prime {\text{v}}_{l}\prime \right]=-\nabla \prime P\prime +\nabla \prime \cdot \left(\mu \prime \nabla \prime {\text{v}}_{l}\prime \right),$$ (2)

in which *ρ'* is the blood density (kg/m^3^), *μ'* is the dynamic viscosity of blood (Pa·s), ${{\text{v}}^{\prime }}_{l}$ is the velocity vector of blood in the lumen (m/s), *P'* is the thermodynamic pressure (Pa) and ∇' is the gradient operator. The prime means a dimensional variable and the absence of a prime indicates a non-dimensional parameter.

The steady-state drug transport was represented in the lumen by

$${\text{v}}_{l}\text{'}\cdot \nabla \prime c=D_{l}\text{'}\nabla \prime^{2}c,$$ (3)

and in the tissue by

$$D_{t}\text{'}\nabla \prime^{2}c=0.$$ (4)

*c* represents the normalised drug concentration, defined as the ratio of the local drug concentration, *c'* (kg/m^3^), to the concentration of drug at the strut surfaces *${c^{\prime }}_{0}$* (kg/m^3^) on which it is assumed to be uniform, viz

$$c=\frac{c^{\prime }}{c_{0}{}^{\prime }},$$ (5)

and *${D^{\prime }}_{l}$* and ${D^{\prime }}_{t}$ represent the diffusivity of the drug in the blood and tissue respectively. Blood is assumed incompressible with a density of *ρ*' = 1060 kg/m^3^. The antiproliferative drug Paclitaxel served as a model compound in this analysis, chosen for its use as the active agent in DES and balloon catheters with a large corpus of data regarding its vascular penetration and transport in blood and within arterial tissue. Its diffusivity coefficients are ${D^{\prime }}_{l}$ = 3.89×10^–11^ m^2^/s [19] and ${D^{\prime }}_{t}$ = 3.65×10^–12^ m^2^/s [20] respectively. A constant diffusivity of drug in blood was assumed, independent of local haematocrit or shear rate, since drug transport was modelled in the boundary layer in which such effects were assumed negligible. Specifically, erythrocytes are not in high concentration in the boundary layer and hence their impact on modulating the drug transport would be small. The flow rates in the boundary layer are also small and therefore assumed to be reasonably measured by a diffusivity measured statically [19]. Furthermore, the global diffusivity of drug in the tissue utilised in this study does not take into consideration the effects of tissue anisotropy and heterogeneity. More complex drug transport models have been described elsewhere which implement negative sink terms to account for drug binding effects in the vascular wall [21]. However, our simplified convection-diffusion model will help to isolate the luminal flow patterns which enhanced or diminished drug uptake.

The finite volume solver ANSYS FLUENT 14.5 (ANSYS Inc.) was used to perform the numerical simulations. A semi-implicit (SIMPLEC) algorithm coupled the pressure and velocity while a second order central differencing scheme spatially discretised the pressure and momentum variables. A second order upwind scheme was also used to discretise the scalar drug concentration.

### Boundary Conditions

The elution of drug from the strut surfaces was modelled using a Dirichlet boundary condition, with *c* = 1. Continuity of flux was assumed at the lumen-tissue interface, while zero concentration, *c* = 0, was imposed at the inlet, implying that blood arrives from the inlet drug free. The walls of large veins and arteries are nourished with blood by a network of fine blood vessels, the “vasa vasorum”. This process of supplying blood to the artery walls can yield one of two boundary conditions at the perivascular wall: either saturation can be achieved, resulting in zero mass flux (*∂c/∂y*′ = 0), or the drug can be completely washed away (*c* = 0) [22]. In this study, the latter condition was assumed to be more realistic as the vasa vasorum are continuously replenished with fresh blood [2]. Finally, zero mass flux of drug was specified on the remaining boundaries.

In order to see the effects of different flow rates on the resulting drug distribution in the tissue, three flow rates were implemented: ${Q^{\prime }}_{mean}$ representing the mean flow during the cardiac cycle, while ${Q^{\prime }}_{high}$ and ${Q^{\prime }}_{low}$ were twice and half ${Q^{\prime }}_{mean}$ respectively. These flow rates were then used in conjunction with each rheological model and a Poiseuille parabolic inlet velocity profile established fully developed flow in each case. A volumetric flow rate of 6.64 mL/s was used for ${Q^{\prime }}_{mean}$, which corresponds to a Reynolds number of 427 under the assumption of a constant dynamic viscosity, *μ'* = 0.00345 Pa·s. These low Reynolds numbers were consistent with the mean flow conditions of the renal vasculature [5] and enabled blood flow to be modelled as laminar in all cases.

All remaining boundary conditions remained the same in each of the simulations performed. A uniform, zero gauge pressure boundary condition was specified at the outlet, whilst no-slip conditions were prescribed on the strut-lumen and lumen-tissue interfaces. A fixed wall assumption was also implemented in light of findings which suggest that stented arteries are considerably stiffer than unstented arteries and that minimal artery motion occurs [23]. Finally, a symmetry boundary condition was specified at the top of the lumen domain.

### Blood Rheology Models

**Fig 2:**
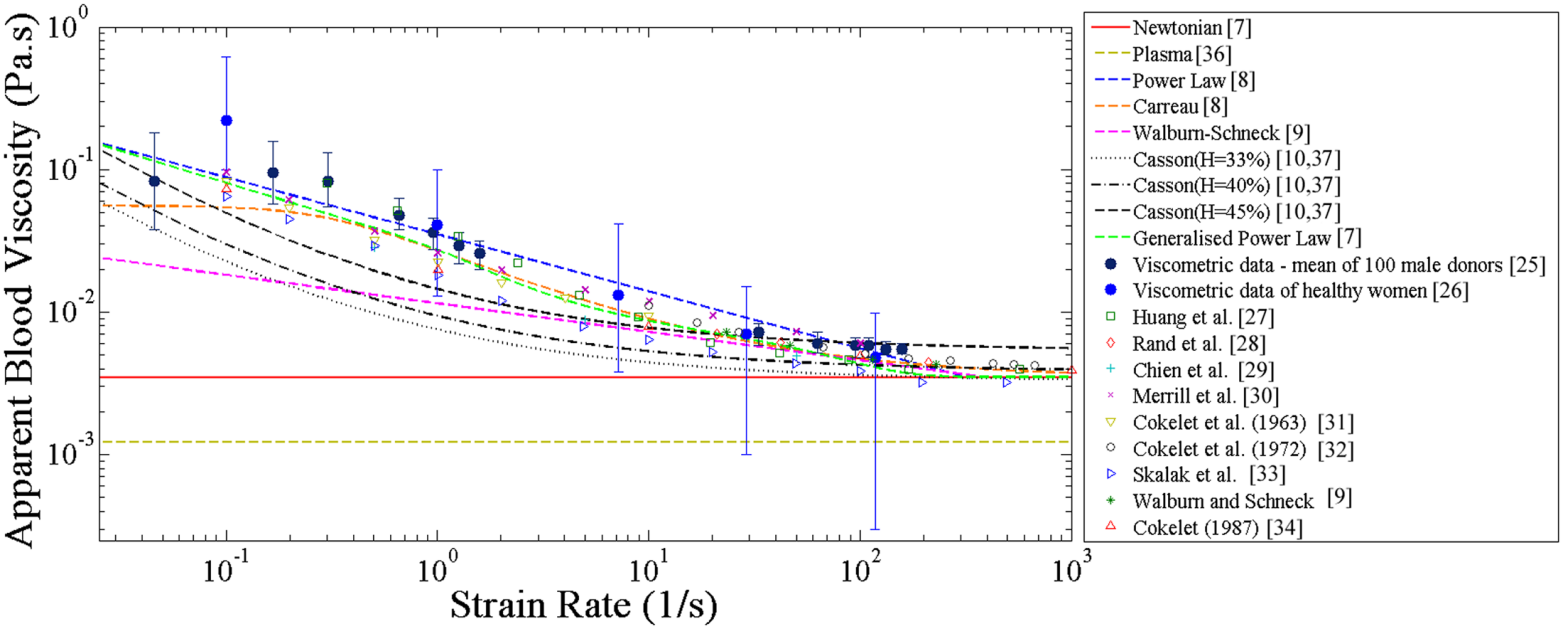
Experimental measurements of blood viscosity and non-Newtonian blood rheological models.

In the Newtonian fluid model, the dynamic viscosity is assumed to remain at a constant value of ${\mu^{\prime }}_{N}$ = 0.00345 Pa·s [7]. Although this assumption greatly simplifies the modelling of blood, it has been found to only be acceptable in flows in which strain rates above 100s^-1^ are encountered [24]. Such high strain rates are found in larger arteries and consequently many investigators have justified their assumption of blood being a Newtonian fluid by emphasising the large size of the arteries that they were modelling [1, 3]. However, the separated flow regions near the stent are characterised by low velocities, meaning that strain rates are modest. As non-Newtonian behaviour may significantly affect velocity distributions in these regions, it may also affect the rate at which drug is removed from the surface and convected by blood. Hence, five non-Newtonian models of blood rheology which incorporated shear- and haematocrit-dependent viscosities were also examined in this study. These models were chosen because they span the spectrum of viscosity variations with respect to strain-rate which have been observed in clinical data (Fig 2 [25–34]). The mathematical formulations of these models are given in Table 1.

**Table 1:** Blood rheological model equations.

Blood Model	Effective Viscosity (Pa·s)
Newtonian [7]	*μ* = 0.00345 *Pa·s*
Plasma [36]	*μ* = 0.00122 *Pa·s*
Power Law (Modified) [8]	$\mu =\{\begin{array}{c} m{\left(\dot{\gamma }\right)}^{n_{P}-1},\;\;\dot{\gamma }<427\\ 0.00345\;Pa\cdot s,\;\;\dot{\gamma }\geq 427 \end{array}$, *m* = 0.035, *n_p_* = 0.6
Walburn-Schneck (Modified) [9]	$\mu =\{\begin{array}{c} C_{1}e^{\left(C_{2}H\right)}e^{\left(C_{4}\left(\frac{TPMA}{H^{2}}\right)\right)}{\left(\dot{\gamma }\right)}^{-C_{3}H},\;\;\dot{\gamma }<414\\ 0.00345\;Pa\cdot s,\;\;\;\dot{\gamma }\geq 414 \end{array}$, C1 = 0.00797, C2 = 0.0608, C3 = 0.00499, C4 = 14.585, H = 40, TPMA = 25.9
Casson [10,37]	$\mu =0.1\left({\left[\sqrt{\eta }+\sqrt{\tau_{y}\left(\frac{1-e^{-m\left|\dot{\gamma }\right|}}{\left|\dot{\gamma }\right|}\right)}\right]}^{2}\right),$ τ_y_ = (0.625H)3, η = η0(1-H)^-2.5^, *η*_0_ = 0.012, H = 40% (female normal), 33% (post-angioplasty) or 45% (male normal)
Carreau [8]	$\mu =\mu_{\infty C}+\left(\mu_{0}-\mu_{\infty C}\right){\left[1+{\left(\lambda \dot{\gamma }\right)}^{2}\right]}^{\frac{n_{c}-1}{2}},$λ = 3.313, *n*_C_ = 0.3568, μ_0_ = 0.056, and μ_∞C_ = 0.00345
Generalised Power Law [7]	$\mu =\lambda {\left|\dot{\gamma }\right|}^{n-1},$ $\lambda =\mu_{\infty \text{G}}+\Delta \mu \text{exp}\left[-\left(1+\frac{\left|\dot{\gamma }\right|}{\text{a}}\right)\text{exp}\left(-\frac{\text{b}}{\left|\dot{\gamma }\right|}\right)\right],\;\;$ $\text{n}={\text{n}}_{\infty }-\Delta \text{n exp}\left[-\left(1+\frac{\left|\dot{\gamma }\right|}{\text{c}}\right)\text{exp}\left(-\frac{\text{d}}{\left|\dot{\gamma }\right|}\right)\right],\;$μ_∞G_ = 0.0035, n_∞_ = 1.0, Δμ = 0.025, Δn = 0.45, a = 50, b = 3, c = 50, and d = 4

The rheological behaviour of blood close to a wall is a particularly complex phenomenon and its viscosity in these regions in not precisely known. In steady, fully-developed flow, red blood cells migrate towards the vessel axis, leaving a plasma-rich region near the walls which is relatively void of red blood cells [35]. We therefore studied the full spectrum of rheology considering the extreme cases of a boundary layer entirely depleted of red blood cells and a boundary layer rich in red blood cells. The fluid properties of plasma (*ρ* = 1025kg/m^3^ and *μ* = 0.00122 Pa·s [36]) were used to approximate the former case whilst the aforementioned Newtonian and non-Newtonian blood rheological models were used to represent the latter case.

As shown in Fig 2, the apparent viscosity predicted by each rheological model is approximately constant for shear strain rates (${\dot{\gamma }}^{\prime }$) greater than 400 s^-1^. As ${\dot{\gamma }}^{\prime }\to 0{\text{s}}^{-1}$ however, each of the non-Newtonian models predicts a different increase in the apparent viscosity of blood, thus demonstrating shear-thinning behaviour, so that at low strain rates they differ significantly from each other and from values obtained from viscometric data which is also presented in Fig 2. Whereas the Carreau model gives a maximum value of the absolute viscosity as the strain rate approaches zero, the viscosities of the Casson, Generalised Power Law, Power Law and Walburn-Schneck models each increase towards infinity. Furthermore, the original formations of the Power Law and Walburn-Schneck models predict zero viscosities as ${\dot{\gamma }}^{\prime }\to \infty {\text{s}}^{-1}$. Although the behaviour of blood as ${\dot{\gamma }}^{\prime }\to 0{\text{s}}^{-1}$ is still debated, zero viscosity at infinite ${\dot{\gamma }}^{\prime }$ is unphysical and hence limitations have been placed on the Power Law and Walburn-Schneck models in order to artificially mimic the Newtonian behaviour of blood at high strain rates. The Generalised Power Law and Carreau model at high strain rates are each approximately asymptotic to the usually used value of the Newtonian model without any need for artificial limitations.

The original Casson model was developed for a yield-pseudo-plastic [30]. This means that unlike a fluid, which is defined by the fact that motion is induced if a shear stress is applied, a yield-pseudo-plastic behaves as if it were a solid if a shear stress less than the yield stress is applied. To alleviate these effects, a modified Casson model was proposed by Razavi *et al*. [37] in which no yield stress is present, and it was this model which was implemented in this current study. This modified Casson model was implemented with three hæmatocrit levels in order to determine how the hæmatocrit affects the haemodynamics and drug transport. The first of these models incorporated a hæmatocrit of 40%, typical of a healthy adult female [11], whilst the second incorporated a lowered, post-angioplasty hæmatocrit level of 33% [12]. Finally, the third Casson model implemented a hæmatocrit of 45%, normal for an adult male [11].

### Grid Description and Refinement

The mesh density used was greatest in the regions closest to the stent strut and also near the interface between the tissue and lumen. This enabled the resolution of thin boundary layers which occurred along the no-slip boundaries defining the artery wall and the stent strut walls. Furthermore, the high mesh density in the tissue and close to the stent strut facilititated the resolution of high drug concentration gradients. This final mesh contained 2,009,929 elements.

Mesh convergence testing was carried out to ensure that the solutions obtained were independent of the size of the grid used. This testing was performed under mean flow conditions using the Newtonian blood rheological model. The flow was deemed to be adequately resolved once the grid convergence index (GCI) corresponding to the recirculation lengths proximal and distal to the stent strut fell below 1%. The GCI is defined as [38]:

$$GCI_{fine\;grid}=\frac{3\left|\frac{{L^{\prime }}_{fine}-{L^{\prime }}_{coarse}}{{L^{\prime }}_{fine}}\right|}{r^{p}-1},$$ (6)

where ${L^{\prime }}_{fine}$ and ${L^{\prime }}_{coarse}$ are the recirculation length proximal or distal to the stent strut for a fine and coarse mesh respectively (mm), *r* is the refinement factor, and *p* is the order of accuracy of the solution. In this case, *r* = $\sqrt{2}$ and *p* = 2. The results of this analysis are listed in Table 2.

**Table 2:** Mesh Independence of the Flow.

Case	No. of Elements	*L*’_*proximal*_ (mm)	GCI	*L*’_*distal*_ (mm)	GCI
1	1,003,861	0.065	-	0.115	-
2	2,009,929	0.065	0%	0.115	0%
3	4,028,702	0.065	0%	0.115	0%

Although the flow was shown to be clearly resolved in each case, the mesh-dependence of the drug-transport behaviour also needed to be evaluated. This was accomplished by comparing the area-weighted average concentration (AWAC) value for each case, which represents the average concentration of drug in a representative area of arterial tissue. This representative area was chosen as that of a rectangle bounded by the upper and lower extents of the tissue domain and the vertical lines *x'* = -0.35mm and *x'* = 0.35mm. This axial extent was chosen on the basis that a typical inter-strut distance is 7 struth widths [1]. Mesh convergence was defined to occur once <2% change was observed between two successive mesh refinements, similar to the drug transport convergence criteria used in prior numerical DES studies [4, 5]. These results are listed in Table 3.

**Table 3:** Mesh Independence of the AWAC Drug Transport Variable.

Case	No. of Elements	AWAC	% change
1	1,003,861	0.156	**–**
2	2,009,929	0.159	1.9%
3	4,028,702	0.160	0.6%

## Results and Discussion

### Rheological Effects on Blood Flow

#### Recirculation Length and Normalised Mean Viscosity

**Fig 3:**
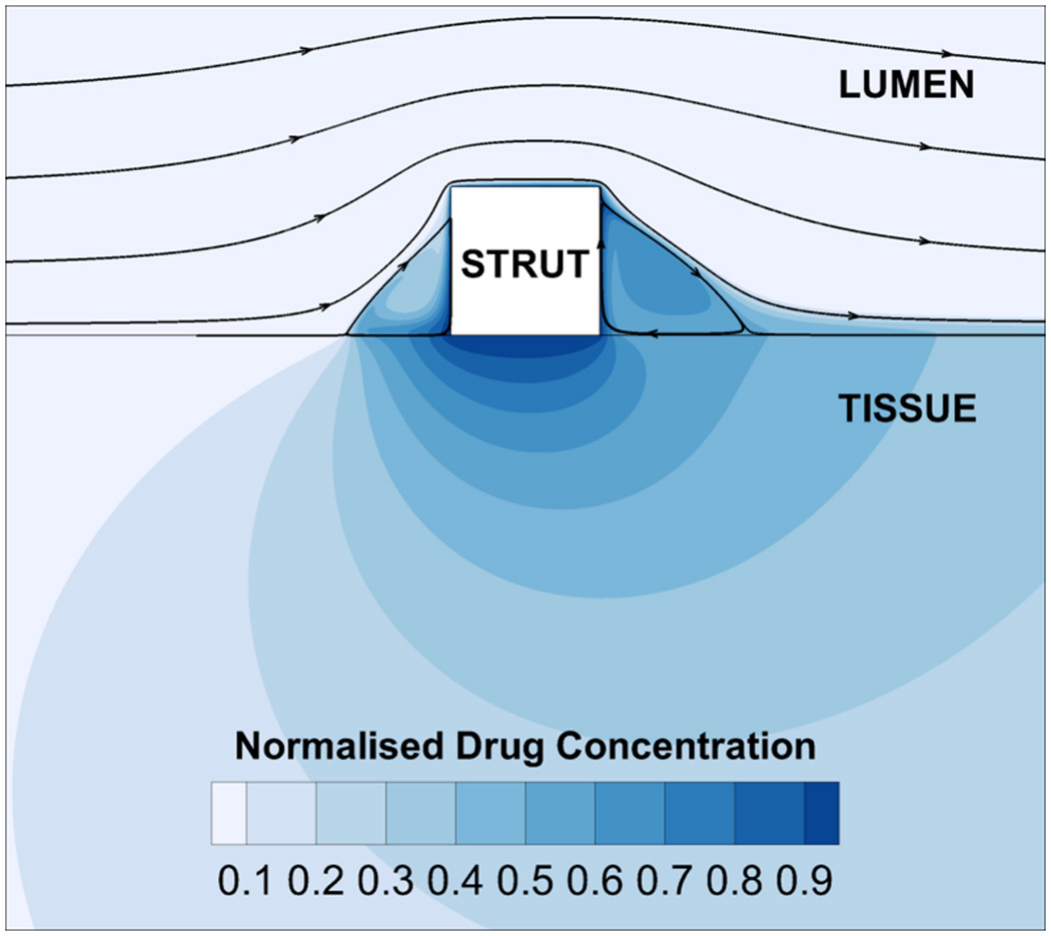
Drug concentration distribution and flow pathlines in the stented artery.

In each steady-state analysis performed, recirculating flow regions were observed to form proximal and distal to the stent strut, and were also observed to correspond with regions of high drug concentration, as found in past DES studies [4, 5]. This phenomenon is shown in Fig 3 for a case employing the Newtonian blood viscosity model at an inlet flow rate of ${Q^{\prime }}_{low}$.

**Fig 4:**
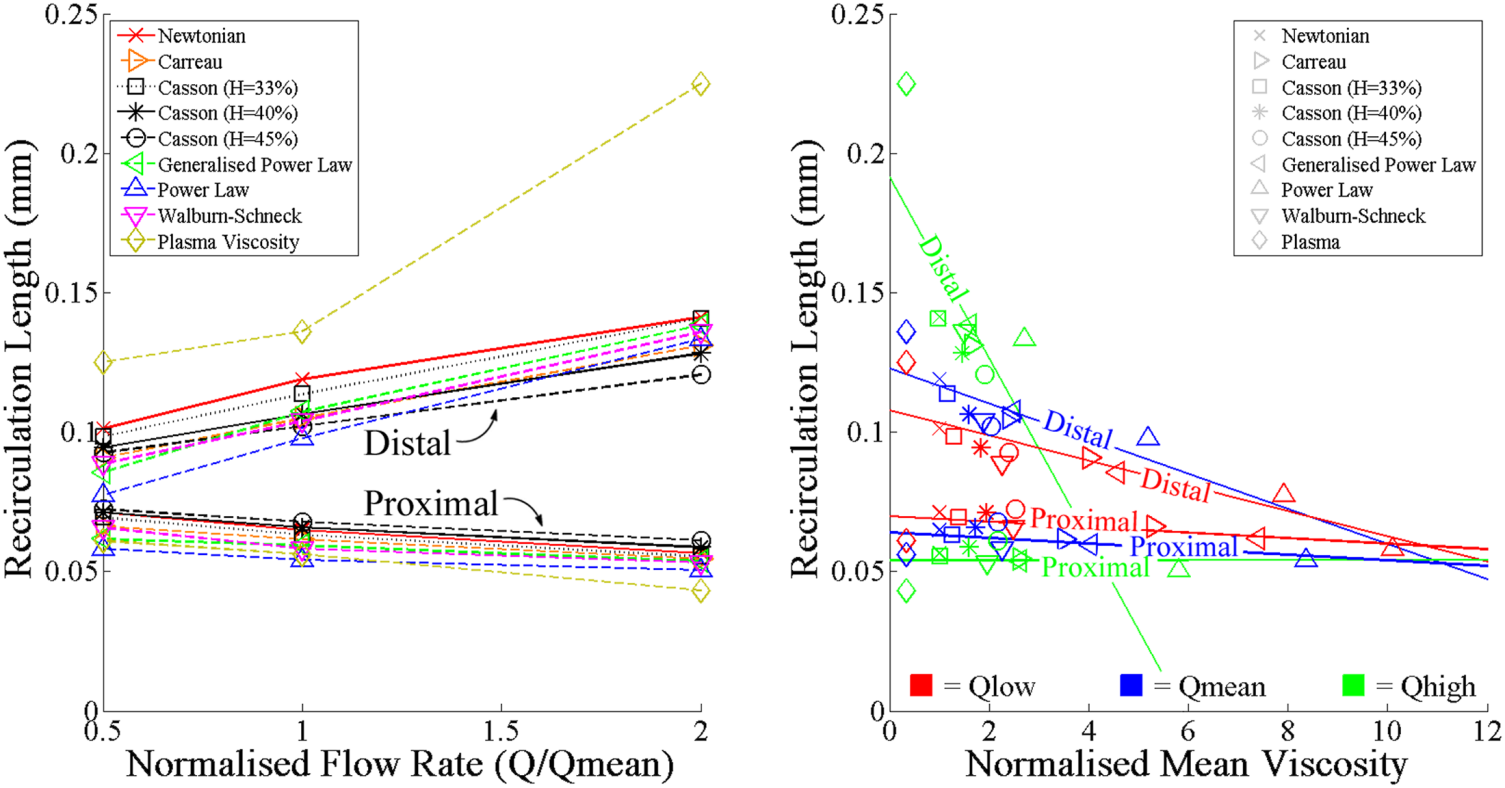
The effects of flow rate and blood rheology on the proximal and distal recirculation lengths. a) Increases in bulk flow rate were found to cause reductions in the proximal recirculation length and increases in the distal recirculation length produced by each rheological model. b) The relationship between recirculation length and normalised mean viscosity, which measures the ratio of the average apparent viscosity in a recirculation zone and the Newtonian model’s dynamic viscosity (${\mu^{\prime }}_{N}$ = 0.00345Pa·s), was found to closely approximate linearity at each inlet flow rate. Furthermore, increases in normalised mean viscosity were found to cause reductions in recirculation length at each flow rate.

The lengths of these recirculating regions were affected by both the flow rate and the choice of blood rheological model, as may be seen in Fig 4a and 4b. Specifically, increases in the flow rate were associated with smaller proximal and larger distal recirculating flow regions for each rheological model investigated. The Newtonian model yielded larger proximal and distal recirculating regions than most of the non-Newtonian models at all flow rates, with the exception of two of the Casson models in the proximal region. In contrast, the Power Law model tended to produce the smallest recirculating flow regions, 18% smaller than the Newtonian model in the proximal region and 24% smaller in the distal region at ${Q^{\prime }}_{low}$. This difference diminished as the flow rate increased, becoming 11% smaller in the proximal region and 6% lower than the Newtonian model in the distal region at ${Q^{\prime }}_{high}$. The Generalised Power Law, Walburn-Schneck and Carreau models produced similar recirculation length results to one another, smaller than the Newtonian model and generally larger than the Power Law model. Each of these non-Newtonian models also tended to converge towards the haemodynamic behaviour of the Newtonian model as the flow rate increased, although this behaviour was less evident in the three Casson models. It was also less evident in the model implementing plasma as the working fluid, which yielded a 24% smaller proximal recirculation length and a 59% larger distal recirculation length at ${Q^{\prime }}_{high}$.

To help ascertain why the non-Newtonian models tended to produce smaller recirculation regions, a new non-dimensional parameter dubbed the *normalised mean viscosity*, $\bar{\bar{\mu }}$, was introduced. This parameter, defined as

$$\bar{\bar{\mu }}=\frac{\int_{A^{\prime }}\mu^{\prime }dA^{\prime }}{A^{\prime }{\mu^{\prime }}_{N}},$$ (7)

measures the average value of the apparent blood viscosity, *μ'* (Pa·s), in the area, *A'* (mm^2^), of the proximal or distal recirculation zone being investigated, normalised by the dynamic viscosity associated with the Newtonian blood rheological model, ${\mu^{\prime }}_{N}$ = 0.00345 Pa·s. As the size of the proximal and distal recirculation zones varied between models and flow rates, the value of *A'* was different in each case investigated.

Despite the significant differences between the rheological behaviour of the different models, the results of Fig 4b showed that the relationship between the recirculation lengths and $\bar{\bar{\mu }}$ approximated linearity at most flow rates. This linear relationship indicates that the elevated viscosities of the non-Newtonian models in the recirculation zones is directly linked to smaller recirculation lengths as the higher viscosities mean that higher stresses are needed to generate the same shear rates so that there is greater resistance to motion. The R^2^ index reveals that these linear trends are most noticeable at ${Q^{\prime }}_{low}$, in which R^2^ = 0.41 in the proximal region and 0.62 in the distal region. However, these values dropped to 0.00 and 0.48 respectively at ${Q^{\prime }}_{high}$. The rheological models most responsible for this loss of linearity were the plasma model and two of the Casson models (H = 40% and 45%). These Casson models produced significantly larger proximal recirculation lengths than the Newtonian model at ${Q^{\prime }}_{high}$, despite having larger $\bar{\bar{\mu }}$ values. Conversely, the plasma model yielded significantly larger distal recirculation lengths than the Newtonian model despite possessing a smaller $\bar{\bar{\mu }}$. The ensuing section outlines how these discrepancies may have transpired.

#### Non-Newtonian Importance Factor

**Fig 5:**
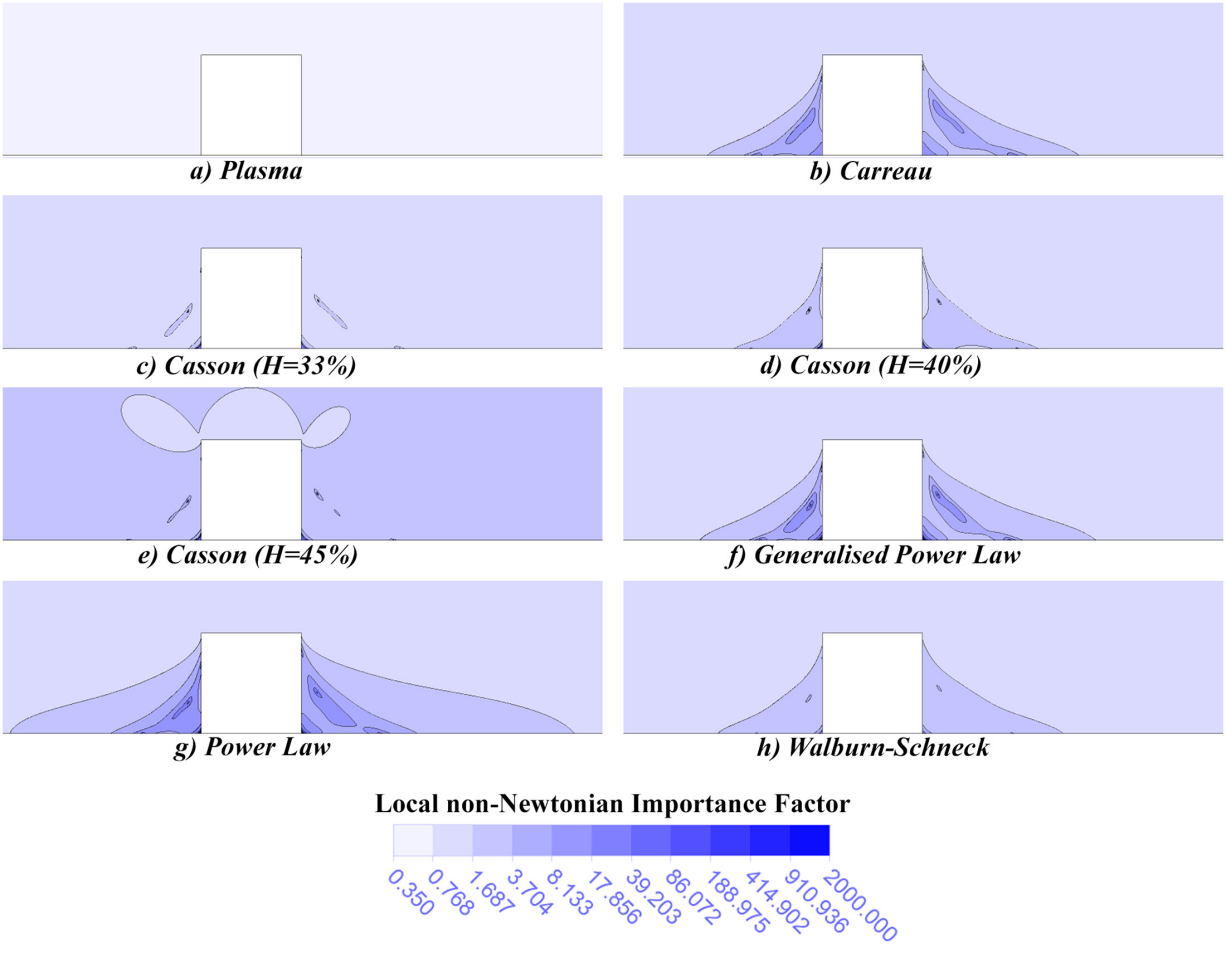
Local non-Newtonian importance factors, *I*_*L*_. This non-dimensional parameter is used to illustrate the spatial variation in non-Newtonian behaviour for each blood rheological model. Although the plasma case (a) yielded a constant value of *I*_*L*_, the remaining cases (b-h) showed that the most significant non-Newtonian behaviour occurs in the recirculation zones.

In order to quantify the significance of non-Newtonian flow behaviour, Ballyk *et al*. [7] introduced a concept referred to as the *non-Newtonian importance factor*:

$$I_{L}=\frac{\mu^{\prime }}{{\mu^{\prime }}_{N}},$$ (8)

where *μ'* is the apparent blood viscosity, while ${\mu^{\prime }}_{N}$ = 0.00345 Pa·s is the dynamic viscosity associated with blood at high strain rates. As non-Newtonian behaviour was generally most pronounced at low flow rates, only the results obtained at the lowest flow rate,${Q^{\prime }}_{low}$, are shown in Fig 5.

The graphs of *I*_*L*_ obtained in this study confirm that the regions of highest dynamic viscosity occurred in both recirculation regions for each non-Newtonian blood rheology model. However, the third Casson model (*H* = 45%) was also found to yield high *I*_*L*_ values in high strain rate regions, such as near the tissue. The high dynamic viscosities found in these regions caused an increased resistance to blood flow, thereby reducing the size of the distal recirculation zone and increasing the size of the proximal zone. This could account for why this model produced significantly larger proximal recirculation lengths than the Newtonian model at each flow rate despite having larger $\bar{\bar{\mu }}$ values, and why it yielded a significantly smaller distal recirculation length than the other models at ${Q^{\prime }}_{high}$.

These results also demonstrated that the magnitude of non-Newtonian behaviour is affected by the hæmatocrit level. The low-hæmatocrit Casson model (*H* = 33%) yielded smaller *I*_*L*_ values than the other Casson models in high and low strain rate regions alike. The associated decrease in resistance to flow in high strain rate regions and in recirculating flow regions resulted in larger distal recirculation lengths. It also yielded smaller proximal recirculation lengths, although the reduced resistance to vortex formation in the proximal recirculating flow region antagonises this behaviour. Hence, this low-hæmatocrit Casson model was found to produce the haemodynamic environment most similar to that of the Newtonian model.

The smallest *I*_*L*_ values were associated with the plasma model, which yielded a constant *I*_*L*_ of 0.35. The associated decrease in resistance to flow in high strain rate regions and in recirculating flow regions resulted in the largest distal recirculation lengths of any of the models examined. It also yielded some of the smallest proximal recirculation lengths, although the reduced resistance to vortex formation in the proximal recirculating flow region antagonised this behaviour somewhat. This antagonistic behaviour could explain why the plasma model’s proximal recirculation lengths remained comparable to the other models at all flow rates whilst its distal lengths differed more dramatically.

### Rheological Effects on Drug Transport

#### Diffusive mass transport across the lumen-tissue interface

Fick’s Law of diffusion states that the diffusive mass transfer across the lumen-tissue interface is proportional to the concentration gradient:

$$\dot{m}\prime =-D_{t}\prime \frac{\partial c\prime }{\partial n\prime }$$ (9)

where ${\dot{m}}^{\prime }$ is the mass flux of the drug species (kg/m^2^s), ${D^{\prime }}_{t}$ is the diffusivity of the drug in the tissue, and ∂*c'*/∂*n'* is the dimensional concentration gradient of drug. *c'* is the concentration of drug (kg/m^3^) and *n'* is the direction normal to the lumen-tissue interface (m), taken positive in the positive *y'* direction. Using the definition of the normalised drug concentration in Eq (5) and non-dimensionalising *n'* in Eq (9), so that

$$n=\frac{n^{\prime }}{{L^{\prime }}_{inter-strut}},$$ (10)

where *L'*_*inter-strut*_ is the same inter-strut distance used in the AWAC calculations (m), a normalised drug concentration gradient parameter was created which reveals the mass transport behaviour at the aforementioned representative section of the lumen-tissue interface as

$$\frac{\partial c}{\partial n}=-\frac{{\dot{m}}^{\prime }{L^{\prime }}_{inter-strut}}{c_{0}{}^{\prime }D_{t}{}^{\prime }}.$$ (11)

**Fig 6:**
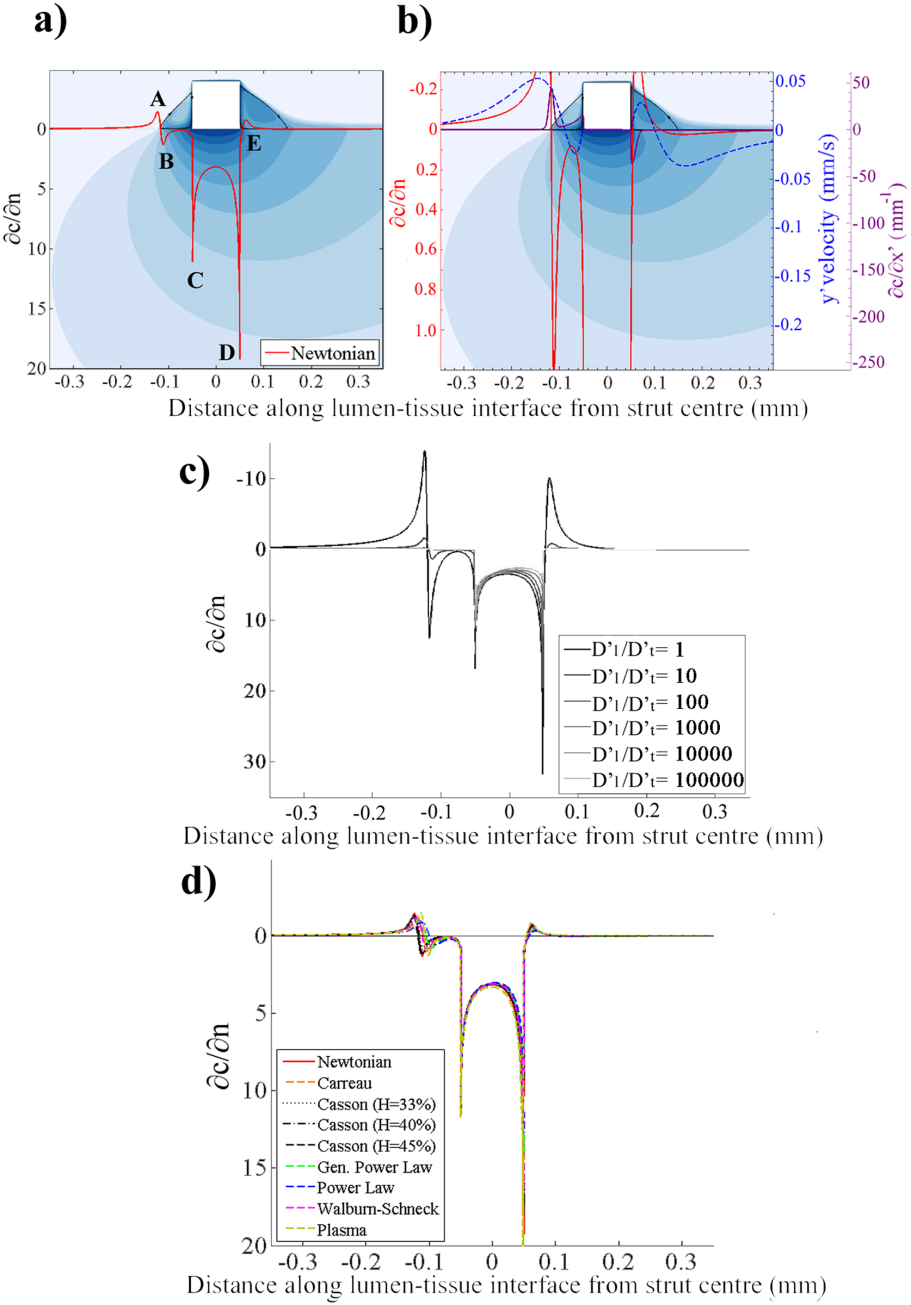
The effect of blood rheological model and flow rate on diffusive mass transport behaviour. a) The plot of the normalised drug concentration gradient for the case in which blood is modelled as a Newtonian fluid revealed five peaks and troughs, labelled A, B, C, D and E. Local minima (A and E) show where drug is removed from the tissue while local maxima (B-D) show where it diffuses into the tissue. b) The dashed blue line shows the distribution of the normal component of the blood velocity along a line 0.1 strut widths above the lumen-tissue interface. Comparison with the red *dc/dn* line confirmed that the upward flow at points A and E resulted in loss of drug from the tissue due to convection. The purple *∂c/∂x*’ line revealed that drug transport in the horizontal direction was significant between points A and B, and at points C and E. c) the distal recirculation zone was found to remove drug from the tissue for non-Paclitaxel drugs, although this behavior diminished as $D_{l}^{'}{/D}_{t}^{'}\geq 100$. d) The non-Newtonian blood rheological models produced similar *dc/dn* patterns to the Newtonian model; however, their local maxima and minima were up to 59% smaller in magnitude. The size of these peaks and troughs was found to be directly related to the recirculation lengths predicted by each model. Hence, the choice of rheological model not only influenced the fluid dynamics, but also the drug transport behaviour.

The normalised drug concentration gradient distribution associated with the Newtonian model in Fig 6a revealed five local peaks and troughs, labelled A, B, C, D and E. Although drug matter was evident in the proximal and distal segments, the concentration gradients at the lumen-tissue interface were only significant in magnitude in the far upstream (points A and B), and downstream (point E) regions. This behaviour could be readily explained by the pooling of drug in the proximal and distal regions driving diffusion processes, and local velocity vectors driving convection processes (Fig 6b). In particular we see at point A, upstream of the proximal recirculation region, low luminal drug concentrations and a velocity vector driving flow into the lumen and giving rise to highly negative ∂*c*/∂*n* values. Closer to the proximal strut surface we see drug concentration gradients of more or less zero, likely due to the low local velocities preventing convection, and a balance in luminal and tissue drug concentration preventing diffusion.

We also noted that the contribution to drug uptake by the distal recirculation zone was inferior to that of the proximal zone. Specifically, integrating ∂*c*/∂*n* along the sections of the lumen-tissue interface occupied by the proximal and distal recirculation regions, we could reveal that the distal zone detracted from the drug transport into the tissue (∫(∂*c*/∂*n*)dx’ = -0.009mm) whilst the proximal zone enhanced drug transport (∫(∂*c*/∂*n*)dx’ = 0.45mm). For the distal region one can see that a larger recirculation region in which the drug can pool would act to dilute the luminal drug concentration, as well as a normal velocity vector (Fig 6b) that acts to drive drug away from the tissue, minimising any convective transport processes. The significance of these results is that they convey that designing haemodynamic stents struts which mitigate this distal recirculation zone may enhance drug uptake.

An additional series of simulations were performed to investigate whether the distal recirculation zone displayed a similar tendency to drive drug out of the tissue with drugs other than Paclitaxel. These simulations were each performed with the Newtonian model at ${Q^{\prime }}_{low}$ and whilst keeping the drug diffusivity in the tissue at a constant value of $D_{t}^{'}$ = 3.65×10^–12^ m^2^/s. The diffusivity in the lumen was varied with each simulation to establish diffusivity coefficient ratios of $D_{l}^{'}{/D}_{t}^{'}$ = 1, 10, 100, 1000, 10000 and 100000. Comparison of the graphs obtained in Fig 6c revealed higher concentration gradients—and hence greater drug transport across the lumen-tissue interface—at lower $D_{l}^{'}{/D}_{t}^{'}$ ratios. Although the drug concentration gradients across the lumen-tissue interface became insignificant as $D_{l}^{'}{/D}_{t}^{'}\geq 100$, a net loss of drug was nonetheless observed underneath the distal recirculation zone irrespective of $D_{l}^{'}{/D}_{t}^{'}$. Hence, the distal recirculation zone does display the tendency to remove drug from the tissue for non-Paclitaxel drugs although this behaviour diminishes as $D_{l}^{'}{/D}_{t}^{'}\geq 100$.

Similar drug concentration gradient profiles to the Newtonian model were similarly observed in the non-Newtonian and plasma cases in Fig 6d as well, although with magnitudes scaled. Particularly, the magnitudes of the peaks and troughs differed by up to 59% from those of the Newtonian model. These differences could be attributed to the fact that a change in the models would act to 1) vary the local velocity—but not to the extent that it would change the balance of convection and diffusion—and 2) vary the relative size of the recirculation regions. Hence the Newtonian model, with its larger recirculation regions, generally yielded the highest magnitude ∂*c*/∂*n* values of the blood models whilst the Power Law model’s smaller recirculation regions yielded the smallest values. The plasma model yielded the highest magnitude ∂*c*/∂*n* values overall, likely because of the high local velocities facilitated by its lower viscosity.

The highest ∂c/∂n values were found at the strut-tissue interface, particularly at the corners at points C and D in each case. Examination of the high magnitude longitudinal concentration gradient (∂c/∂x’) values at these points in Fig 6b revealed that large quantities of drug were removed longitudinally from beneath the strut. It was this removal of drug beneath the strut which facilitated the high ∂c/∂n values between points C and D and especially at the strut corners themselves. A similar phenomenon was observed at point B, which also featured high magnitude ∂c/∂x’ values due to the loss of drug upstream, facilitating a local ∂c/∂n peak. A higher magnitude ∂c/∂n was observed at point D than at point C due to the velocity vector aft of point D which drives drug out of the tissue (and thereby enhances the concentration gradient in the y’ direction). The effects of flow and blood rheological model selection on the drug transport are further outlined in the ensuing sections.

#### Area-Weighted Average Concentration

**Fig 7:**
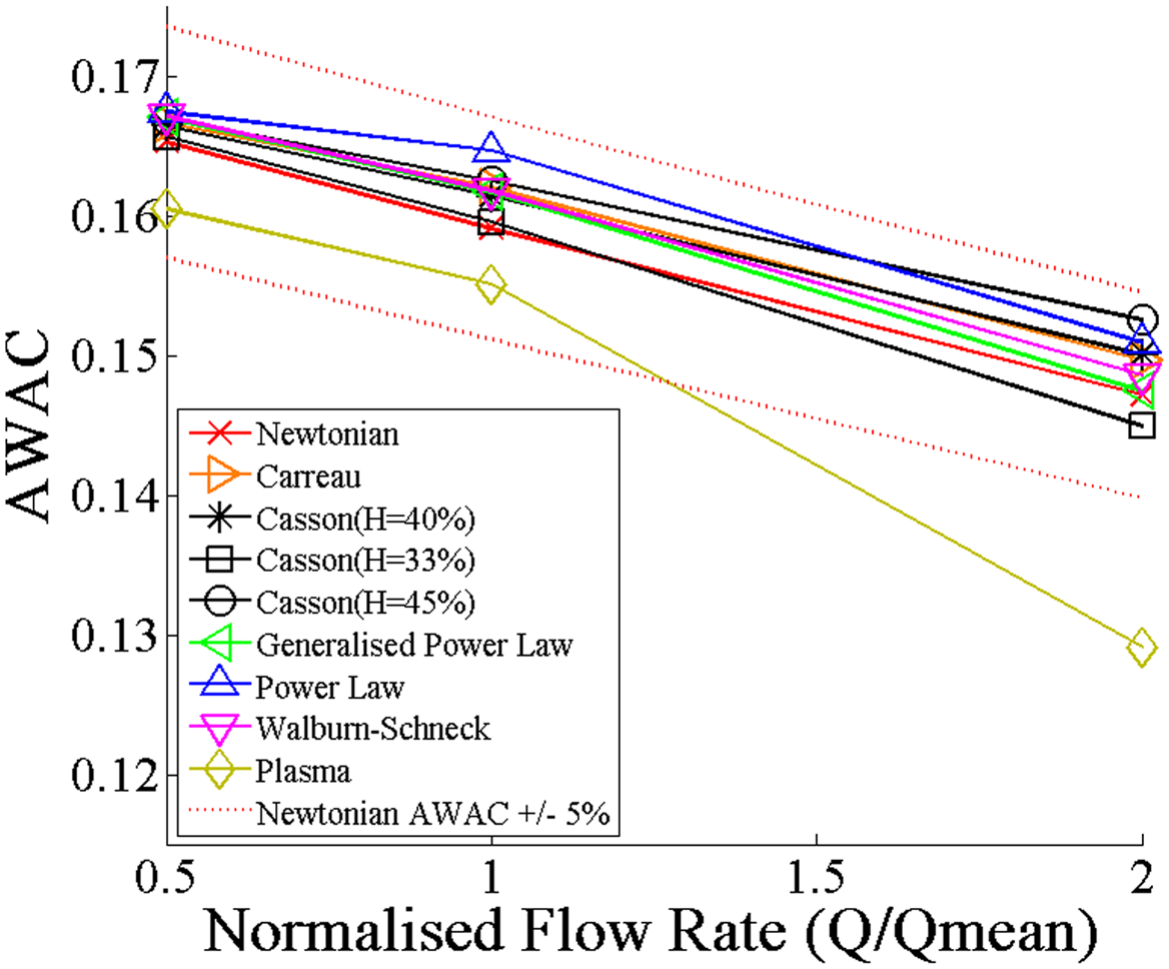
The effect of blood rheological model and flow rate on the average tissue drug concentration. Increases in bulk flow rate were found to correspond with reduced area-weighted average drug concentrations (AWAC) in the tissue. Rheological models which produced larger recirculation lengths were also found to produce lower AWAC values; however, the deviation from the AWAC obtained with the Newtonian model was observed to be less than 5% for each non-Newtonian case. It was only when modelling plasma instead of blood that any considerable deviations from the Newtonian model’s AWAC were observed and even these were only observed at ${Q^{\prime }}_{high}$.

The AWAC of drug in the arterial tissue was dependent on both the flow rate and on the choice of the blood rheological model. An inverse relationship was found to exist between the AWAC and the flow rate, and, more precisely, between the AWAC and the sizes of the proximal and distal recirculating flow regions. The Newtonian model and the post-angioplasty Casson model (H = 33%), with their larger recirculation zones, therefore tended to produce the lowest AWAC of each of the blood rheological models at each flow rate while the Power Law model generally yielded the highest AWAC, as shown in Fig 7. However, these AWAC values deviated less than 5% from those of the Newtonian model at all of the flow rates tested. In fact, it was only when modelling plasma instead of blood that any considerable deviations from the Newtonian model’s AWAC were observed and even these only occurred at ${Q^{\prime }}_{high}$. This suggests that the Newtonian model is appropriate to use in place of non-Newtonian blood rheological models in studies seeking to quantify the magnitude of arterial drug uptake. The clinical significance of these results is that they convey that the magnitude of drug uptake in stent-based drug delivery is relatively invariant of individual variations in blood rheology. However, transient simulations implementing pulsatile inlet velocity profiles may be needed to confirm whether or not the Plasma model’s AWAC deviates significantly from the Newtonian model’s over several cardiac cycles.

Although these results appear to convey that the Newtonian model is appropriate for use in DES studies, they have neglected to account for the effects of blood rheology on the spatial distribution of drug in the tissue. This investigation and its outcomes are outlined in the ensuing section.

#### Non-Newtonian Drug Concentration Difference Factor

Another new parameter being introduced in this study is the *non-Newtonian drug concentration difference factor*, *I*_*D*_, and it represents the difference between the normalised drug concentration of the concerned non-Newtonian rheological model and the Newtonian model at the same flow rate, divided by the integrated absolute concentration gradient along a representative length of the lumen tissue interface:

$$I_{D}=\frac{c_{NN}\;-c_{N}}{\int_{-0.35mm}^{0.35mm}\left|\frac{\partial c_{N}}{\partial n\prime }\right|dx\prime }.$$ (12)

As earlier, this representative length is chosen to lie between the points *x'* = -0.35mm and *x'* = 0.35mm along the line *y'* = 0mm. The subscripts *NN* and *N* indicate non-Newtonian and Newtonian cases respectively.

**Fig 8:**
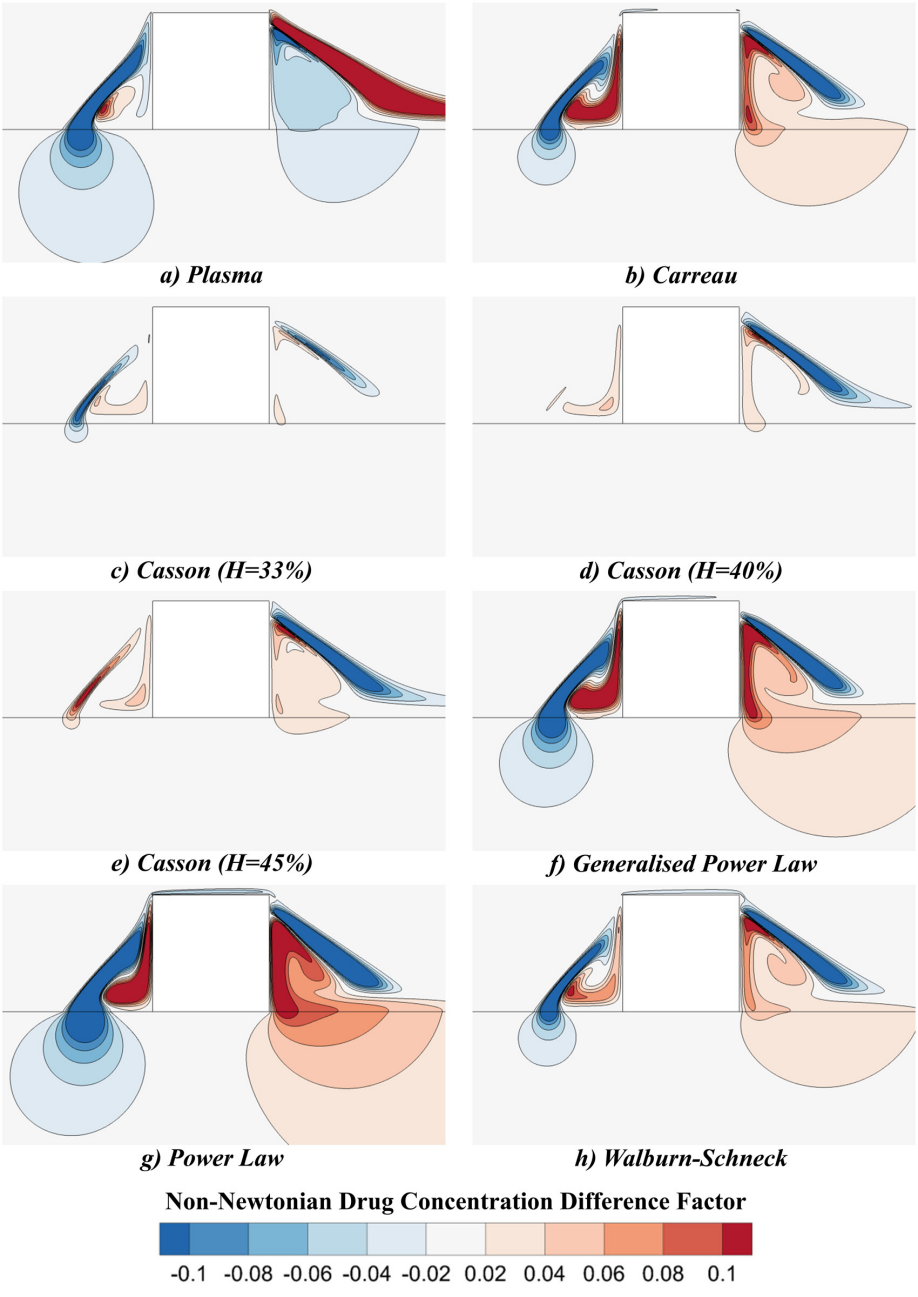
Contour plots of the non-Newtonian drug concentration difference factor, *I*_*D*_. These contours display the degree to which a rheological model’s predicted drug concentration departs from that associated with the traditional Newtonian model. The regions highlighted in red (*I*_*D*_>0) depict where the non-Newtonian blood model predicts a greater drug concentration, whilst the blue regions (*I*_*D*_<0) show where the Newtonian model predicts a higher drug concentration. The plasma case (a) is different from the other cases examined in that its larger distal recirculation zone resulted in a less concentrated distal drug pool than that of the Newtonian model. This larger pool allowed a greater region of the lumen-tissue interface to be exposed to recirculating drug; however, no significant positive *I*_*D*_ values were observed in the tissue. In contrast, the larger proximal drug pool of the Newtonian model did facilitate significant negative *I*_*D*_ values in the proximal sections of the tissue. In contrast, cases b-h show that the smaller recirculation lengths of the non-Newtonian models enable the formation of higher concentration drug pools than the Newtonian model. Although significant negative *I*_*D*_ values were again observed in the proximal aspects of the tissue, significant positive *I*_*D*_ values were also observed in the distal tissue aspects in some models. The non-Newtonian blood rheological models therefore typically produced much higher tissue drug concentrations than the Newtonian model in the distal regions and significantly lower concentrations in the proximal regions.

The effect of blood rheology on the distribution of drug in the artery wall was ascertained by a comparison of the *I*_*D*_ plots at the three flow rates investigated. The results of this study are shown in Fig 8. Once again, only the results corresponding with a flow rate of ${Q^{\prime }}_{low}$ are displayed, as this is where non-Newtonian effects were generally most pronounced. The regions highlighted in red (*I*_*D*_>0) depict where the non-Newtonian blood model predicts a greater drug concentration, whilst the blue regions (*I*_*D*_<0) show where the Newtonian model predicts a higher drug concentration. Regions in which |*I*_*D*_| >0.06 were deemed to correlate with regions in which the non-Newtonian model’s spatial distribution of drug matter departed significantly from that of the Newtonian model.

Although the Newtonian model was deemed adequate for predicting the AWAC, the presence of regions with high |*I*_*D*_| established that non-Newtonian effects were significant when investigating the spatial distribution of drug matter in arteries featuring DES. The drug pools which formed in the smaller recirculating flow regions generally associated with the non-Newtonian models were significantly more concentrated than those of the Newtonian models, as conveyed by the red areas found immediately proximal and distal to the stent struts in Fig 8. Although the smaller recirculation regions of the non-Newtonian models did facilitate higher concentration drug pools, the strain rates associated with the Newtonian model were greater in the regions of the recirculation zones which were close to the lumen-tissue interface. These higher strain rates facilitated an increased convective transport of drug and may account for why *I*_*D*_ became negligibly small near the proximal lumen-tissue interface in each case, despite the higher concentrations of the drug pools in the non-Newtonian cases. *I*_*D*_ did not become negligible, however, at the distal lumen-tissue interface as the drug matter which reached the interface was already significantly more dilute in the Newtonian case. The result of these effects is that the Newtonian model tended to yield higher tissue drug concentrations upstream of the strut whilst the non-Newtonian models produced higher concentrations in the downstream region. The combination of these effects could account for why the AWAC values of the non-Newtonian models deviated less than 5% from that of the Newtonian model despite the significant differences in drug spatial distribution which were observed.

Although a non-Newtonian blood rheological model may more accurately describe the spatial distribution of drug in arterial tissue, it is difficult to convey which model is best suited to this task. Comparison of the results obtained with the three Casson models revealed that patients with higher haematocrits may yield higher drug uptake globally but particularly in regions distal to the stent struts. However, as the Power Law model typically yielded the most significant non-Newtonian behaviour, it is suggested that both Newtonian and Power Law models be implemented in future studies concerned with drug transport details. This method may be used to determine a range of potential drug transport behaviours and thus be of potential use to stent designers. A plasma model may also be appropriate to incorporate on the basis of the relatively low tissue drug concentrations that it yields in both upstream and downstream tissue aspects. However, transient simulations implementing a pulsatile inlet velocity may be needed to confirm if these results depart significantly with those achieved with the Power Law and Newtonian models over several cardiac cycles.

## Conclusions

Non-Newtonian effects were generally most pronounced at low flow rates and the choice of blood rheological model was found to influence flow patterns and drug transport. The largest non-Newtonian haemodynamic and drug transport effects were observed in the Power Law model, while these effects were more modest in cases employing the Walburn-Schneck, Casson, Carreau and Generalised Power Law models. These non-Newtonian effects typically manifested through significantly reduced proximal and distal recirculation lengths when compared with the Newtonian model. An additional blood plasma model was also implemented to account for red blood cell depletion in the near-wall regions. This model yielded smaller proximal and larger distal recirculation lengths than the Newtonian model.

In each model investigated, the flow separation regions which formed downstream of the stent struts were found to diminish the drug uptake. These results, when considered in conjunction with relevant experimental data, could lead to the design of more haemodynamic DES struts which mitigate this distal recirculation zone and potentially enhance drug uptake.

Numerical methods allowed us to appreciate the subtle but still significant differences in drug delivery due to blood rheology. We found that non-Newtonian effects can be significant and the choice of a non-Newtonian rheological model is contextually important. Specifically, a Newtonian model was found to be appropriate to use in studies seeking to quantify the magnitude of arterial drug uptake, although non-Newtonian effects were found to impact the spatial distribution of drug in the tissue. It was therefore suggested that both Newtonian and Power Law rheological models be implemented in future numerical studies concerned with drug transport details, in order to establish a range of potential drug concentration distributions. A plasma model may also be appropriate to incorporate on the basis of its relatively small tissue concentrations in both proximal and distal regions.

Clinically, these results conveyed that the magnitude of drug uptake in stent-based drug delivery is relatively invariant of individual variations in blood rheology. Furthermore, it was also suggested that patients with higher haematocrits may yield higher drug concentrations globally but particularly in regions distal to the stent struts. As our understanding of near-wall viscosities is limited however, an in-depth discussion of which rheological models to use in specific cases (e.g. females, males, post-surgery) was deemed beyond the scope of the current study.

## References

[1] Balakrishnan B, Tzafriri AR, Seifert P, Groothuis A, Rogers C, Edelman ER. Strut position, blood flow, and drug deposition: implications for single and overlapping drug-eluting stents. Circ. 2005; 111: 2958–2965. 15927969 10.1161/CIRCULATIONAHA.104.512475

[2] Mongrain R, Faik I, Leask RL, Rodés-Cabau J, Larose E, Bertrand OF. Effects of diffusion coefficients and struts apposition using numerical simulations for drug eluting coronary stents. J Biomech Eng. 2007; 129: 733–742. 17887899 10.1115/1.2768381

[3] Zunino P, D'Angelo C, Petrini L, Vergara C, Capelli C, Migliavacca F. Numerical simulation of drug-eluting coronary stents: mechanics, fluid dynamics and drug release. Comput Methods Appl Mech Eng. 2009; 198: 3633–3644.

[4] Kolachalama VB, Tzafriri AR, Arifin DY, Edelman ER. Luminal flow patterns dictate arterial drug deposition in stent-based delivery, J. Control. Release. 2009; 133: 24–30. 10.1016/j.jconrel.2008.09.075 18926864 PMC2836846

[5] O'Brien CC, Kolachalama VB, Barber TJ, Simmons A, Edelman ER. Impact of flow pulsatility on arterial drug distribution in stent-based therapy. J. Control. Release. 2013; 168: 115–124. 10.1016/j.jconrel.2013.03.014 23541929 PMC3697861

[6] Mejia J, Mongrain R, Bertrand OF. Accurate prediction of wall shear stress in a stented artery: Newtonian versus non-Newtonian models. J. Biomech. Eng. 2011; 133: 074501 10.1115/1.4004408 21823750

[7] Ballyk PD, Steinmann DA. Simulation of non-Newtonian blood flow in an end-to-end anastomosis. Biorheol. 1994; 31; 565–586. 10.3233/bir-1994-31505 7833458

[8] Cho YI, Kensey KR. Effects of the non-Newtonian viscosity of blood on flows in a diseased arterial vessel. Part 1: steady flows. Biorheol. 1991; 28: 241–262. 10.3233/bir-1991-283-415 1932716

[9] Walburn FJ and Schneck DJ. A constitutive equation for whole human blood, Biorheol. 1976; 13: 201–210. 10.3233/bir-1976-13307 953256

[10] Fung YC. Biomechanics: mechanical properties of living tissues. 2nd ed Boston: Springer; 1993.

[11] Billett HH. Hemoglobin and Hematocrit In: Walker HK, Hall WD and Hurst JW, editors. Clinical methods: the history, physical, and laboratory examinations. 3rd ed Boston: Butterworths; 1990. 21250045

[12] Phillips SJ, Spector M, Zeff RH, Skinner JR, Toon RS, Grignon A and Kongtahworn C. Hematocrit changes after uncomplicated percutaneous transluminal coronary angioplasty. Am J Cardiol. 1989; 64: 940 2641673 10.1016/0002-9149(89)90848-5

[13] Khan W, Farah S and Domb AJ, Drug eluting stents: developments and current status. J Control Release. 2012; 161: 703–712. 10.1016/j.jconrel.2012.02.010 22366546

[14] Van Jaarsveld BC, Krijnen P, Pieterman H, Derkx FHM, Deinum J, Postma CT, et al The effect of balloon angioplasty on hypertension in atherosclerotic renal-artery stenosis. N Engl J Med. 2000; 342: 1007–1014. 10749962 10.1056/NEJM200004063421403

[15] White CJ, Ramee SR, Collins TJ, Jenkins JS, Escobar A, Shaw D. Renal artery stent placement: utility in lesions difficult to treat with balloon angioplasty. J Am Coll Cardiol. 1997; 30: 1445–1450. 9362400 10.1016/s0735-1097(97)00348-3

[16] Rocha-Singh K, Jaff MR, Rosenfeld K. ASPIRE-2 trial investigators evaluation of the safety and effectiveness of renal artery stenting after unsuccessful balloon angioplasty: the ASPIRE-2 study. J Am Coll Cardiol. 2005; 46: 776–783. 16139124 10.1016/j.jacc.2004.11.073

[17] Dorros G, Prince C, Mathiak L. Stenting of a renal artery stenosis achieves better relief of the obstructive lesion than balloon angioplasty. Cathet Cardiovasc Diagn. 1993; 29: 191–198. 8402841 10.1002/ccd.1810290304

[18] Kolachalama VB, Levine EG, Edelman ER. Luminal Flow Amplifies Stent-Based Drug Deposition in Arterial Bifurcations. PLoS ONE. 2009; 4: e8105 10.1371/journal.pone.0008105 19956555 PMC2781163

[19] Lovich MC, Creel CJ, Hong K, Hwang CW and Edelman ER. Carrier proteins determine local pharmacokinetics and arterial distribution of paclitaxel. J Pharm Sci. 2001; 90: 1324–1335. 11745785 10.1002/jps.1085

[20] Creel CJ, Lovich MA and Edelman ER. Arterial paclitaxel distribution and deposition. Circ Res. 2000; 86: 879–884. 10785510 10.1161/01.res.86.8.879

[21] Lovich MA, Edelman ER. Computational Simulations of Local Vascular Heparin Deposition and Distribution. Am J Physiol. 1996; 271: H2014–H2024. 8945921 10.1152/ajpheart.1996.271.5.H2014

[22] Finkelstein A, McClean D, Kar S, Takizawa K, Varghese K, Baeck N, Park K, Fishbein MC, Makkar R, Litvack F, Eigler NL. Local Drug Delivery via a Coronary Stent with Programmable Release Pharmacokinetics. Circulation. 2003; 107: 777–784. 12578884 10.1161/01.cir.0000050367.65079.71

[23] Henry FS. Flow in stented arteries In: Vendonck P, Perktold K, editors. Intra and Extra Corporeal Cardiovascular Fluid Dynamics. Southhampton, UK: WIT Press; 2000.

[24] Berger SA and Jou LD. Flow in stenotic vessels. Annu Rev Fluid Mech. 2000; 32: 347–382.

[25] Dintenfass L. Blood Viscosity. Lancaster: MTP Press Ltd; 1985.

[26] Dintenfass L, Julian DG, Miller G. Viscosity of Blood in Healthy Young Women: Effect of Menstrual Cycle, Lancet. 1966; 288: 234–235. 10.1016/s0140-6736(66)90053-5 4159072

[27] Huang C, King R, Copley A. Rheogoniometric studies of whole human blood at shear rates down to 0.0009s^-1^. Part ii. Mathematical Interpretation. Biorheol. 1973; 10: 17–22. 10.3233/bir-1973-10104 4724174

[28] Rand P, Lacombe E, Hunt H, Austin W. Viscosity of normal human blood under normothermic and hypothermic conditions. J Appl Physiol. 1964; 19: 117–122. 14104265 10.1152/jappl.1964.19.1.117

[29] Chien S, Usami S, Taylor M, Lundberg J, Gregersen M. Effects of hematocrit and plasma proteins on human blood rheology at low shear rates. J Appl Physiol. 1966; 21: 81–87. 5903948 10.1152/jappl.1966.21.1.81

[30] Merrill E, Gilliland E, Cokelet G, Shin H, Britten A, Wells R. Rheology of human blood, near and at zero flow: Effects of temperature and hematocrit level. Biophys J. 1963; 3: 199–213. 13935042 10.1016/s0006-3495(63)86816-2 PMC1366440

[31] Cokelet G, Merrill E, Gilliland E, Shin H, Britten A and Wells R. The rheology of human blood measurement near and at zero shear rate. Trans. Soc. Rheol. 1963; 7: 303–317.

[32] Cokelet G. Biomechanics: Its foundation and objectives. Englewood Cliffs, New Jersey: Prentice-Hall; 1973.

[33] Skalak R, Keller S and Secomb T Mechanics of blood flow, J Biomech Eng. 1981; 103: 102–115. 7024641 10.1115/1.3138253

[34] Cokelet G. The rheology and tube flow of blood In: Skalak R, Chien S, editors. Handbook of Bioengineering. New York: McGraw-Hill; 1987 pp. 14.1–14.17.

[35] Aarts PA, Van Den Broek SA, Prins GW, Kuiken GD, Sixma JJ, Heethaar RM. Blood platelets are concentrated near the wall and red blood cells, in the center in flowing blood. Arteriosclerosis. 1988; 8; 819–24. 3196226 10.1161/01.atv.8.6.819

[36] Pedersen L, Nielsen EB, Christensen MK, Buchwald M, Nybo M. Measurement of plasma viscosity by free oscillation rheometry: imprecision, sample stability and establishment of a new reference range. Ann Clin Biochem. 2014; 51: 495–8. 10.1177/0004563213504550 24081187

[37] Razavi A, Shirani E, and Sadeghi M. Numerical simulation of blood pulsatile flow in a stenosed carotid artery using different rheological models. J Biomech. 2011; 44: 2021–2030. 10.1016/j.jbiomech.2011.04.023 21696742

[38] Roache PJ. Quantification of uncertainty in computational fluid dynamics, Annu Rev Fluid Mech. 1997; 29: 123–160.

